# Rehmannioside A alleviates neuroinflammation and cognitive impairments after traumatic brain injury by suppressing microglial activation via the MAPK/NF-κB pathway

**DOI:** 10.3389/fneur.2026.1788639

**Published:** 2026-05-13

**Authors:** Shiyu Zhou, Yiwan Fang, Haoxin Ji, Huazheng Yan, Jianxiong Gao, Hezuo Lü

**Affiliations:** Department of Immunology, Bengbu Medical College, Anhui Key Laboratory of Infection and Immunity at Bengbu Medical University, Bengbu, Anhui, China

**Keywords:** MAPK/NF-κB pathway, microglia, neuroinflammation, Rehmannioside A, traumatic brain injury

## Abstract

**Background:**

Traumatic brain injury (TBI) triggers a robust neuroinflammatory response characterized by microglial activation, which propagates secondary neuronal damage and contributes to long-term neurological deficits. Rehmannioside A (REA), a principal bioactive compound from *Rehmannia glutinosa*, has emerged as a candidate for neuroprotection due to its anti-inflammatory properties. However, its therapeutic potential and precise mechanisms of action in TBI remain to be fully elucidated.

**Methods:**

We employed a controlled cortical impact (CCI) model in mice to mimic clinical TBI. Animals were randomized into Sham/Veh, Sham/REA, TBI/Veh, and TBI/REA (40 mg/kg) groups. Neurological and cognitive functions were assessed using the modified Neurological Severity Score (mNSS) and Morris Water Maze (MWM). Cerebral edema was measured, and histopathological changes were evaluated by H&E and Nissl staining. LPS-stimulated BV2 microglial cells were used for *in vitro* experiments. Pro-inflammatory cytokines were measured by enzyme-linked immunosorbent assay (ELISA) and qRT-PCR, and activation of the MAPK/NF-κB pathway was analyzed by western blotting.

**Results:**

REA treatment significantly improved neurological scores, spatial learning and memory, and reduced cerebral edema and neuronal loss in TBI mice. REA suppressed microglial activation *in vivo* and dose-dependently inhibited LPS-induced pro-inflammatory mediators *in vitro*. These beneficial effects are associated with reduced phosphorylation of p65 (NF-κB) and p38 (MAPK) in activated microglia *in vitro*.

**Conclusion:**

REA ameliorates functional deficits and neuropathology following TBI. The neuroprotective effect may involve suppression of microglia-mediated neuroinflammation via inhibition of the MAPK/NF-κB signaling pathway.

## 1 Introduction

Traumatic brain injury (TBI), resulting from external mechanical forces to the head, represents a major cause of mortality and long-term disability worldwide, posing a significant global public health challenge (1–3). It is clinically characterized by a spectrum of reversible or irreversible neurological impairments. The pathological cascade of TBI unfolds in two distinct phases: primary and secondary injury (4). Primary injury results from immediate mechanical damage caused directly by external force, including cerebral contusions and lacerations, vascular rupture, and physical disruption of the blood-brain barrier. This phase occurs instantaneously, presents significant challenges for clinical intervention, and treatment focuses primarily on sustaining vital functions and preventing further damage. In contrast, secondary injury is a dynamically evolving process that occurs from hours to weeks or even months after the initial insult. Its core consists of a cascade of pathological events including neuroinflammatory responses, disruption of calcium homeostasis, oxidative stress damage, and programmed cell death (5). The pathological alterations in this phase provide a critical time window for clinical intervention. Among the various mechanisms of secondary injury, neuroinflammation is considered a key driver of disease progression (6). Microglia, the resident innate immune cells of the central nervous system, become rapidly activated following TBI (7). Under physiological conditions, microglia are primarily responsible for immune surveillance and clearing cellular debris, thereby maintaining intracranial environmental stability (8). However, after TBI, microglia often exhibit a state of overactivation, shifting to a pro-inflammatory phenotype (M1 type) and releasing a barrage of cytotoxic mediators such as TNF-α, IL-1β, IL-6, and reactive oxygen species (9). This sustained inflammatory response exacerbates damage to the neurovascular unit and promotes neuronal dysfunction and death, creating a vicious cycle that hinders recovery (10). Consequently, strategies aimed at modulating microglial activation and curbing this excessive neuroinflammatory response have emerged as a promising therapeutic avenue for TBI.

Despite this understanding, clinical management of TBI remains predominantly supportive, focusing on symptom control and intracranial pressure management (11). A critical translational gap exists, as no specific therapies are yet available to effectively target maladaptive neuroinflammation and thereby improve functional outcomes (12, 13). This unmet medical need underscores the importance of developing novel, mechanism-based treatments.

Natural products, with their multi-target profiles and favorable safety, offer a valuable source for drug discovery (14). Rehmannioside A (REA), an iridoid glycoside derived from *Rehmannia glutinosa*, has shown preliminary neuroprotective and anti-inflammatory potential in models of spinal cord injury, reportedly by modulating microglial polarization toward an anti-inflammatory phenotype (15, 16). However, its efficacy and precise mechanism of action in TBI remain unexplored (17).

Therefore, this study was designed to systematically evaluate the therapeutic potential of REA in TBI and to elucidate its underlying molecular mechanisms. Using an integrated approach combining a mouse controlled cortical impact model and LPS-stimulated BV2 microglia, we investigated whether REA could improve functional recovery, reduce cerebral edema, and attenuate neuronal loss and neuroinflammation *in vivo*. Furthermore, we focused on the NF-κB and MAPK signaling pathways—key regulators of inflammation—as potential mechanistic targets for REA. Our findings aim to provide a solid experimental foundation for positioning REA as a promising candidate for the treatment of TBI and other neuroinflammatory conditions.

## 2 Materials and methods

### 2.1 Animals and experimental design

A total of 40 8-week-old female C57BL/6 mice [weighing 20–22 g, specific pathogen-free (SPF) grade] were obtained from Shanghai Model Organisms Center and housed in the Experimental Animal Center of Bengbu Medical College [License No.: SYXK (Wan) 2023-009] under controlled conditions (temperature: 22–26 °C; humidity: 45–65%; 12-h light/dark cycle) with *ad libitum* access to food and water. All experimental procedures were approved by the Animal Ethics Committee of Bengbu Medical College (Approval No.: 2020-050) and conducted in accordance with AAALAC guidelines. The mice were randomly divided into four groups (*n* = 10 per group). The sham + vehicle group underwent scalp incision and craniectomy without cortical impact, followed by daily intraperitoneal injections of 0.2 mL vehicle. The sham + REA group received identical surgical procedures followed by daily injections of 40 mg/kg REA. The TBI + vehicle group was subjected to controlled cortical impact (CCI) injury followed by daily vehicle injections. The TBI + REA group underwent CCI injury followed by daily 40 mg/kg REA treatment. REA was dissolved in DMSO and diluted in normal saline to achieve the final concentration, with all injections maintained at 0.2 mL volume. REA (40 mg/kg) or vehicle was administered intraperitoneally immediately after surgery, once daily until 24 h before sample collection. The treatment duration was 1–21 consecutive days depending on experimental endpoints. All mice in the same assay received the same dosing scheduleMice were randomly allocated using a random number table. All behavioral and histological assessments were performed by investigators blinded to group assignments. REA was dissolved in DMSO and diluted in normal saline (final DMSO concentration <0.1%).

### 2.2 Traumatic brain injury model

The traumatic brain injury model was established using a standardized controlled cortical impact (CCI) device. After anesthetizing the mice with an intraperitoneal injection of ketamine (80 mg/kg) and xylazine (10 mg/kg), each animal was securely positioned in a stereotaxic frame under aseptic conditions. A midline scalp incision was made to expose the skull, and a 3.5-mm diameter craniotomy was performed centered at coordinates 2.0 mm posterior and 1.5 mm lateral to the bregma. The dura mater remained intact throughout the procedure. Cortical impact was delivered using a 3-mm diameter impactor tip at a velocity of 4 m/s to a depth of 1.5 mm below the cortical surface (18). Sham-operated animals underwent the same surgical procedure including scalp incision and craniotomy but did not receive cortical impact. Following surgery, the scalp was sutured closed, and animals were placed on a 37 °C heating pad for recovery with continuous monitoring of vital signs until full awakening.

### 2.3 Modified Neurologic Severity Score (mNSS)

Spatial learning and memory were assessed using the Morris Water Maze (MWM) system at post-operative days 1, 3, 7, 14, and 21 (19). The test was performed at 2:00 p.m. under consistent environmental conditions by a single investigator, with data analyzed in a double-blinded manner. The paradigm included two phases: Spatial acquisition phase: Mice were trained to find the submerged platform on days 1, 3, 7, 14, and 21, and escape latency was recorded as an index of spatial learning ability. Spatial probe trial: On post-operative day 21, the platform was removed, and mice were allowed 60 seconds of free exploration. The percentage of time in the target quadrant, number of platform location crossings, and swimming trajectories were automatically recorded by Smart 3.0 software to evaluate spatial memory retention. To minimize motor confounding effects, only mice with stable locomotor ability were included in the final analysis.

### 2.4 Brain water content

Cerebral edema was quantified by measuring brain water content using the standard wet-dry weight method. At 24 h post-TBI, mice were anesthetized and transcardially perfused with normal saline to remove intravascular blood. Whole brain tissues were rapidly harvested, and the cerebellum and brainstem were carefully dissected away. After gently blotting residual surface moisture with sterile filter paper, the cortical tissue containing hippocampal and thalamic regions was immediately weighed to obtain wet weight (recorded with 0.1 mg accuracy). Samples were then dehydrated in a constant-temperature oven at 100 ± 1 °C for 72 h until stable dry weight was achieved. Brain water content was calculated as: [(wet weight – dry weight) wet weight] × 100%. Throughout the procedure, tissue integrity was meticulously maintained to avoid mechanical compression or artificial damage.

### 2.5 Morris Water Maze (MWM) Test

Spatial learning and memory were assessed using the MWM system. Mice underwent testing at 2:00 p.m. on post-operative days 1, 3, 7, 14, and 21. The evaluation consisted of two phases: a 5-day spatial acquisition phase during which mice were trained to locate a submerged platform, with escape latency recorded as an indicator of spatial learning ability; followed by a spatial probe trial where the platform was removed and animals were allowed 60 s of free exploration. During the probe trial, the percentage of time spent in the target quadrant, number of platform location crossings, and swimming trajectories were automatically recorded using Smart 3.0 software to evaluate memory retention (20). All tests were conducted by the same operator under consistent environmental conditions to ensure reproducibility, and data analysis was performed in a double-blinded manner to prevent observer bias.

### 2.6 Histological staining

Histopathological evaluation was performed seven days post-intervention using standardized perfusion-fixation protocols (21). Following deep anesthesia, mice underwent transcardial perfusion initiated with phosphate-buffered saline (PBS) until effluent from the right atrium cleared, followed by 4% paraformaldehyde (PFA) infusion until limb rigidity was observed. Brains were carefully extracted and sequentially dehydrated in 15%–30% sucrose gradients. After embedding in OCT compound, coronal sections were prepared at 4 μm thickness using a −20 °C cryostat. Tissue morphology was examined through hematoxylin and eosin (H&E) staining, while neuronal survival was quantitatively assessed via toluidine blue Nissl staining. The density of viable neurons was expressed as Nissl-positive cells per square millimeter (cells/mm^2^). For quantification, 5 non-overlapping fields were analyzed per section, 3 sections per mouse, and averaged per animal. All analyses were performed using ImageJ in a blinded manner.

### 2.7 Immunofluorescence

Immunofluorescence staining was performed on post-TBI day 7 to analyze microglial activation. Brain tissues were fixed in 4% paraformaldehyde for 24 h, processed through ethanol dehydration and paraffin embedding, and sectioned at 6 μm thickness (22). After baking at 60 °C for 2 h and antigen retrieval, sections were permeabilized with 0.2% Triton X-100 (15 min, room temperature) and blocked with 5% normal goat serum (Gibco, USA) at 37 °C for 1 h. Sections were then incubated with primary antibody (goat anti-IBA1 polyclonal antibody) overnight at 4 °C, followed by PBS washes and incubation with corresponding secondary antibody at 37 °C for 2 h in the dark. Nuclei were counterstained with DAPI (Beyotime, China) for 5 min, and sections were mounted with anti-fade mounting medium (Vector Laboratories, USA). All images were acquired using a ZEISS Axio observer microscope (Carl Zeiss, Germany), with IBA1-positive cells quantified from five randomly selected fields per group. For quantification, 5 non-overlapping fields were analyzed per section, 3 sections per mouse, and averaged per animal. All analyses were performed using ImageJ in a blinded manner.

### 2.8 RT-qPCR

Total RNA was extracted from both the peri-lesional brain tissue of TBI mice and cultured BV2 microglial cells using TRIzol reagent. RNA purity and concentration were measured using a spectrophotometer. Following reverse transcription using a commercial cDNA synthesis kit, quantitative real-time PCR was performed under the following conditions: denaturation at 95 °C for 30 s, followed by 40 cycles of 95 °C for 5 s and 60 °C for 30 s (23). All reactions were run in triplicate using a SYBR Green detection system. GAPDH served as the endogenous control, and relative mRNA expression levels of IL-1β, IL-6, and TNF-α were calculated using the 2^−Δ*ΔCt*^ method. Primer sequences used in this study are listed in Table 1.

**Table 1:** Primers utilized in this study.

Gene	Primers	Sequence
TNF-α	Forward primers	GTCTACTGAACTTCGGGGTGATC
	Reverse primers	TCCTCCACTTGGTGGTTTGTGA
IL-6	Forward primers	TCTATACCACTTCACAAGTCGGA
	Reverse primers	GAATTGCCATTGCACAACTCTTT
IL-1β	Forward primers	GAAATGCCACCTTTTGACAGTG
	Reverse primers	TGGATGCTCTCATCAGGACAG
GAPDH	Forward primers	TGGCCTTCCGTGTTCCTAC
	Reverse primers	GAGTTGCTGTTGAAGTCGCA

### 2.9 Cell culture

BV2 microglial cells were cultured in high-glucose Dulbecco's Modified Eagle Medium (DMEM) supplemented with 10% heat-inactivated fetal bovine serum and 1% penicillin/streptomycin, and maintained at 37 °C in a humidified 5% CO_2_ incubator (24). For experimental procedures, cells were seeded at consistent densities in 96-well plates and allowed to adhere completely. The cells were divided into three experimental groups: the control group (CON) received no treatment; the LPS group was stimulated with 1 μg/mL lipopolysaccharide to establish an inflammatory model; and the LPS+REA group was pretreated with 1 μg/mL LPS followed by administration of 40 μM REA for 24 h. All experiments were conducted using cells within passages 15–20 to ensure phenotypic consistency.

### 2.10 CCK-8 assay

Cell viability was assessed using the Cell Counting Kit-8 (CCK-8) assay. BV2 microglial cells were seeded in 96-well plates at a density of 1 × 104 cells per well (*n* = 5 replicates per group) and allowed to adhere. After establishing an inflammatory model by stimulating cells with 1 μg/mL LPS for 6 h, the cells were treated with varying concentrations of REA (0–80 μmol/L) for 24 h. Subsequently, 10 μL of CCK-8 solution was added to each well containing 90 μL of fresh DMEM medium, followed by incubation at 37 °C in the dark for 1 h. The optical density of each well was measured at 450 nm using a microplate reader (25). Cell viability was calculated as the percentage relative to the control group absorbance. The experiment was independently repeated three times to ensure reproducibility.

### 2.11 Enzyme-linked immunosorbent assay (ELISA)

The secretion levels of pro-inflammatory cytokines (TNF-α, IL-6, and IL-1β) in BV2 cell culture supernatants were quantified using commercial enzyme-linked immunosorbent assay (ELISA) kits according to the manufacturer's protocols (26). Briefly, BV2 cells were seeded in 6-well plates with five replicate wells per group. After 48 h of culture, supernatants were collected and centrifuged at 1,000 × g for 10 min at 4 °C to remove cellular debris. Standards and samples were then processed following the kit instructions, and absorbance was measured at 450 nm using a microplate reader. Each assay included appropriate standard and blank controls, and all experiments were repeated three times independently under identical conditions to ensure reliability.

### 2.12 Western blotting

Protein expression was analyzed by western blotting following standard protocols (27). Briefly, tissue samples and cultured BV2 cells were lysed in RIPA buffer, and the lysates were centrifuged at 12,000 × g for 15 min at 4 °C to collect supernatants. Protein concentrations were determined using a BCA assay kit. Equal amounts of protein (20–40 μg) from each sample were separated by 12.5% SDS-PAGE and electrophoretically transferred to PVDF membranes. After blocking with 5% non-fat milk for 2 h at room temperature, the membranes were incubated overnight at 4 °C with primary antibodies against iNOS, TNF-α, IL-1β, p-NF-κB p65, NF-κB p65, p38, p-p38, and GAPDH. Following incubation with appropriate HRP-conjugated secondary antibodies for 1.5 h at room temperature, protein bands were visualized using an enhanced chemiluminescence detection system. Band intensities were quantified with ImageJ software, normalized to GAPDH as the loading control. All experiments were independently repeated at least three times to ensure reproducibility.

### 2.13 Statistical analysis

All quantitative data obtained from at least three independent experiments are presented as mean ± standard deviation (Mean ± SD). The normal distribution of data was verified using the Shapiro-Wilk test. For comparisons between two groups, an unpaired Student's *t*-test was applied. For comparisons across multiple groups and repeated time points, two-way repeated-measures ANOVA was employed, followed by Tukey's *post-hoc* test. For comparisons between two groups, an unpaired Student's *t*-test was applied. All statistical analyses were performed using GraphPad Software, LLC, San Diego, CA, United States. A *p*-value of less than 0.05 (*p* < 0.05) was considered statistically significant.

## 3 Results

### 3.1 Rehmannioside A ameliorates cerebral edema and improves cognitive and motor function in a mouse model of TBI

**Figure 1:**
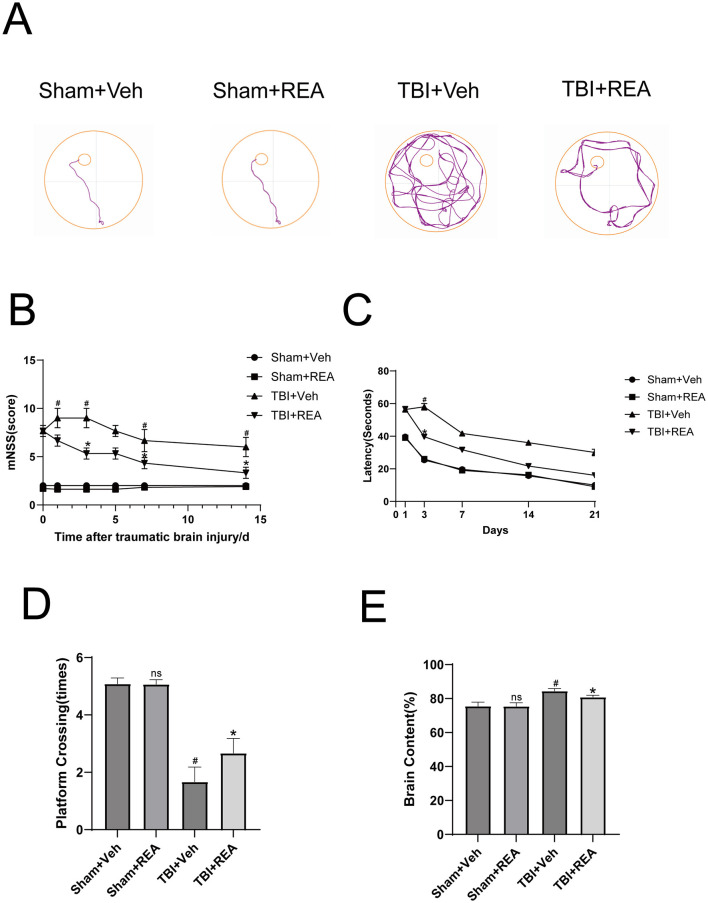
REA treatment improves cognitive and motor function and reduces cerebral edema in TBI mice. **(A)** Representative swimming trajectories from the Morris Water Maze (MWM) probe test. **(B)** The mNSS showed that REA treatment significantly improved neurological function scores compared to the TBI+Veh group. **(C)** REA treatment significantly shortened the escape latency in MWM training trials. **(D)** REA treatment significantly increased the number of platform crossings in the MWM probe test. **(E)** REA treatment significantly attenuated TBI-induced increase in brain water content at 24 h post-injury. Values are expressed as mean ± SEM. *n* = 6 per group. ^#^*P* < 0.05, ^##^*P* < 0.01 vs. Sham+Veh group *P < 0.05, **P < 0.01 vs. TBI+Veh group (two-way repeated-measures ANOVA for behavioral data; one-way ANOVA for others).

To evaluate the impact of Rehmannioside A (REA) on TBI-induced neurological deficits, we first assessed motor and cognitive function. Neurological severity was measured using the modified Neurological Severity Score (mNSS), which revealed significantly higher scores in the TBI+Veh group compared to the Sham+Veh group across all post-injury time points (days 1, 3, 5, 7, and 14). Treatment with REA, however, significantly reduced these scores (TBI+REA vs. TBI+Veh, *P* < 0.05), indicating a notable improvement in neurological function (Figure 1B). Spatial learning and memory were further examined using the MWM. Representative swimming trajectories from the probe trial are shown in Figure 1A. TBI resulted in significant cognitive impairment, manifested as prolonged escape latency during training and a decreased number of platform crossings in the probe trial. Administration of REA markedly ameliorated these deficits, significantly shortening escape latency and increasing the number of platform crossings (Figures 1C, D, *P* < 0.05). We next investigated whether the observed functional improvements were associated with a reduction in cerebral edema. Assessment of brain water content at 24 h post-TBI showed a significant increase in the TBI+Veh group relative to the Sham+Veh group. This finding was supported by histopathological examination of H&E-stained sections. Importantly, REA treatment significantly attenuated the extent of cerebral edema (Figure 1E, *P* < 0.05), suggesting its role in preserving blood-brain barrier integrity and reducing tissue swelling.

### 3.2 REA alleviates histopathological damage and increases neuronal survival in TBI mice

**Figure 2:**
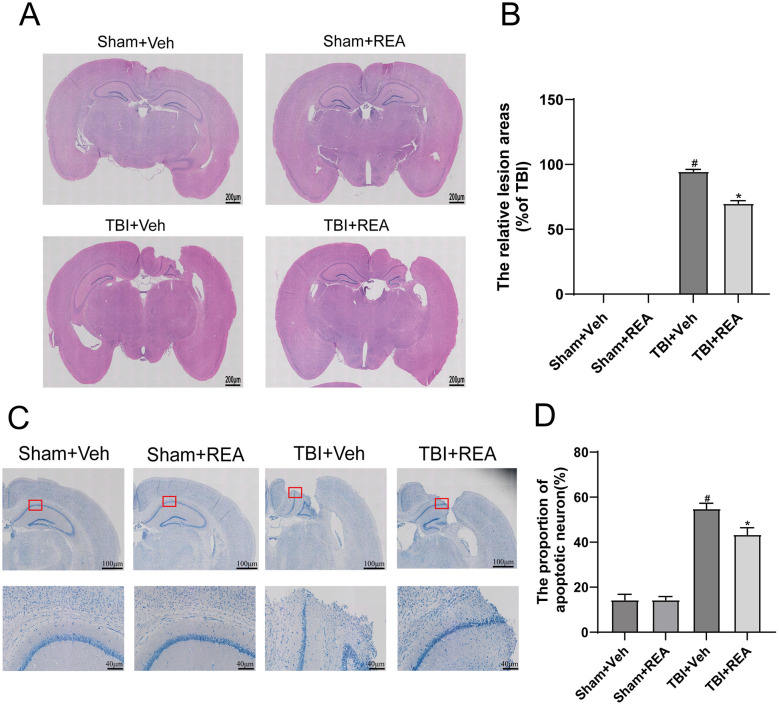
REA confers neuroprotection against TBI-induced histopathological impairment. **(A)** H&E staining shows the hippocampal lesion. Note the more well-preserved architecture in the REA-treated group. **(B)** Quantitative assessment of the damaged tissue area confirms the protective effect of REA. **(C)** Nissl staining reveals neuronal integrity in the hippocampus. **(D)** Quantitative analysis demonstrates a significant increase in neuronal survival with REA treatment, indicating notable neuroprotection. Values are expressed as mean ± SEM. *n* = 6 per group. ^#^*P* < 0.05, ^##^*P* < 0.01 vs. Sham+Veh group *P < 0.05, **P < 0.01 vs. TBI+Veh group (two-way repeated-measures ANOVA for behavioral data; one-way ANOVA for others).

To determine the neuroprotective potential of REA against structural brain damage, we first examined histopathological changes in the hippocampal region. Hematoxylin and eosin (H&E) staining revealed substantial tissue damage in the TBI+Veh group, characterized by cavitation, cellular infiltration, and loss of normal architecture. Quantification of the lesion area demonstrated a significant increase in the TBI+Veh group compared to the Sham+Veh group. Importantly, treatment with REA significantly reduced the extent of this damage (Figures 2A, B, *P* < 0.05). We next assessed neuronal survival using Nissl staining. Consistent with the H&E findings, the number of Nissl-positive cells was markedly decreased in the hippocampal region of the TBI+Veh group, indicating extensive neuronal loss. In contrast, REA treatment markedly increased the number of surviving neurons compared to the TBI+Veh group (Figures 2C, D, *P* < 0.05), further demonstrating its potent neuroprotective effect.

### 3.3 REA suppresses microglial activation and neuroinflammation after TBI

**Figure 3:**
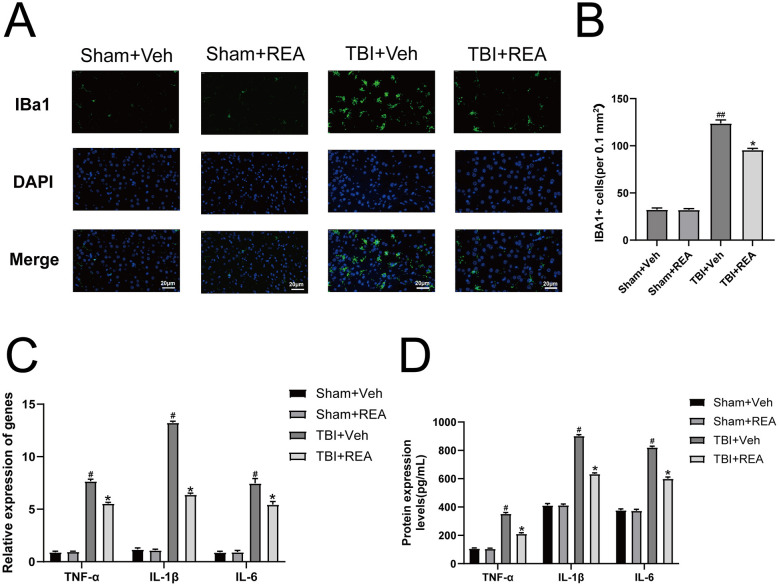
REA mitigates neuroinflammation by inhibiting microglial activation and the subsequent pro-inflammatory cascade. **(A)** Representative images showing microglial morphology and activation. **(B)** Quantification confirms REA significantly reduced the proportion of activated microglia. **(C)** REA intervention suppressed the transcriptional upregulation of key pro-inflammatory mediators. **(D)** Consistent suppression of cytokine release at the protein level by REA was confirmed by ELISA. Values are expressed as mean ± SEM. *n* = 6 per group. ^#^*P* < 0.05, ^##^*P* < 0.01 vs. Sham+Veh group *P < 0.05, **P < 0.01 vs. TBI+Veh group (two-way repeated-measures ANOVA for behavioral data; one-way ANOVA for others).

To investigate whether the neuroprotective effects of REA were associated with the modulation of neuroinflammation, we first assessed microglial activation. Immunofluorescence staining for IBA1 showed a robust increase in the number of activated microglia with amoeboid morphology in the peri-lesion cortex of the TBI+Veh group compared to the Sham group. Quantitative analysis confirmed a significantly higher proportion of activated IBA1-positive cells following TBI. Notably, REA treatment significantly attenuated this microglial activation (Figures 3A, B, *P* < 0.05). We next examined the expression of key pro-inflammatory mediators. qRT-PCR analysis revealed that the mRNA levels of TNF-α, IL-6, and IL-1β were markedly upregulated in the brain tissue of the TBI+Veh group. Intervention with REA significantly suppressed the transcriptional expression of these inflammatory cytokines (Figure 3C, *P* < 0.05). Furthermore, to confirm these findings at the protein level, we performed ELISA. Consistent with the gene expression data, the protein concentrations of TNF-α, IL-6, and IL-1β were significantly elevated in the TBI+Veh group, and this increase was effectively reversed by REA treatment (Figure 3D, *P* < 0.05). Collectively, these results demonstrate that REA effectively inhibits TBI-induced microglial activation and subsequent neuroinflammatory response.

### 3.4 REA suppresses the release of inflammatory mediators in LPS-stimulated microglia

**Figure 4:**
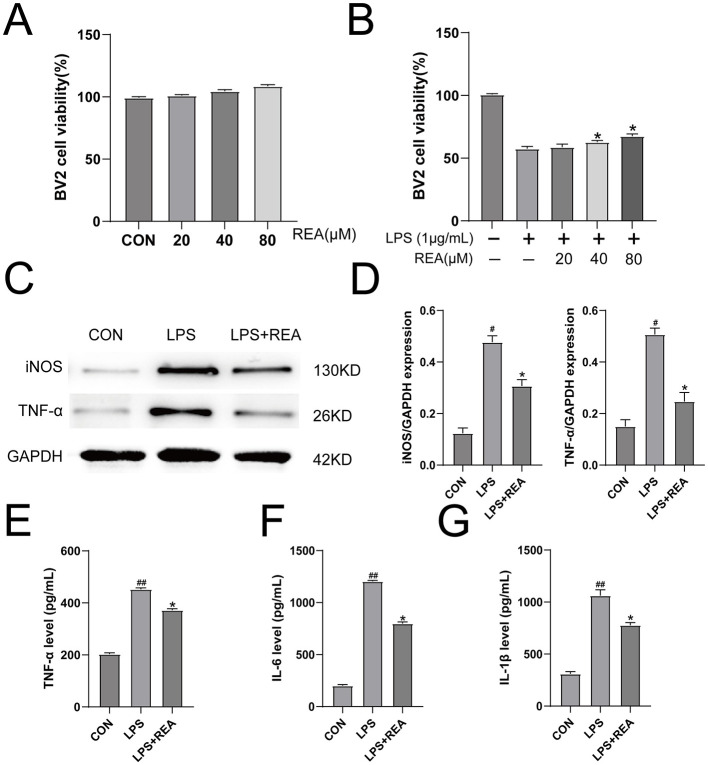
REA exhibits cytocompatibility and concentration-dependent anti-inflammatory effects in activated microglia. **(A)** Assessment of REA cytocompatibility in BV2 cells across a concentration gradient. **(B)** REA conferred protection against LPS-induced cytotoxic injury in a concentration-dependent manner. **(C, D)** The anti-inflammatory effect of REA at the protein level, as evidenced by reduced expression of iNOS and TNF-α. **(E–G)** Functional suppression of cytokine release by REA, validating its role in inhibiting the pro-inflammatory cascade. Values are expressed as mean ± SEM. *n* = 6 per group. **^#^*P* < 0.05, ^##^*P* < 0.01 vs. CON group; *P < 0.05, *P* < 0.01 vs. LPS group (two-way repeated-measures ANOVA for behavioral data; one-way ANOVA for others).

To evaluate the anti-inflammatory effects of REA in a cellular model of neuroinflammation, we first assessed its potential cytotoxicity on BV2 microglial cells. The CCK-8 assay showed that REA, at concentrations ranging from 0 to 80 μmol/L, did not exert any significant effect on BV2 cell viability (Figure 4A), indicating good biocompatibility within this dose range. We next examined whether REA could protect microglia from LPS-induced injury. After stimulating BV2 cells with 1 μg/mL LPS for 6 h, a significant reduction in cell viability was observed. However, treatment with 40 and 80 μmol/L REA significantly attenuated this LPS-induced cytotoxicity (Figure 4B, *P* < 0.05). To further investigate the underlying mechanism, we analyzed the expression of key inflammatory proteins by western blot. The results demonstrated that LPS stimulation markedly upregulated the protein levels of iNOS and TNF-α, whereas treatment with 40 μmol/L REA significantly suppressed their expression (Figures 4C, D, *P* < 0.05). Consistent with these findings, ELISA results confirmed that REA intervention also significantly inhibited the secretion of pro-inflammatory cytokines, including TNF-α, IL-1β, and IL-6, in the culture supernatant of LPS-stimulated BV2 cells (Figures 4E–G, *P* < 0.05). Based on these comprehensive results, 40 μmol/L was selected as the optimal concentration of REA for subsequent mechanistic experiments.

### 3.5 REA exerts anti-inflammatory effects by suppressing the MAPK/NF-κB signaling pathway

**Figure 5:**
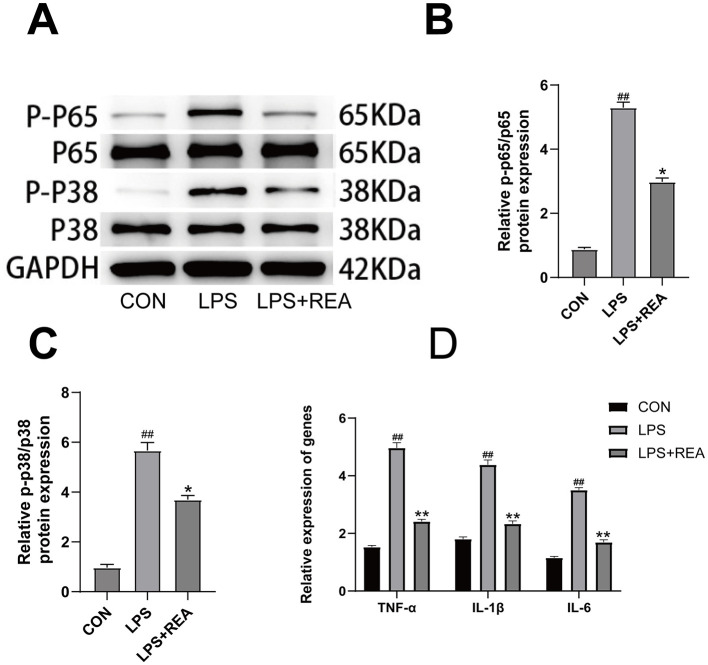
REA mitigates microglial activation by targeting the MAPK/NF-κB signaling axis. **(A)** Western blot analysis of pathway activation. **(B)** Quantification shows REA significantly inhibited NF-κB activation via p65 phosphorylation. **(C)** Quantification shows REA significantly suppressed MAPK activation via p38 phosphorylation. **(D)** The inhibition of upstream signaling by REA resulted in the reduced transcriptional activation of pro-inflammatory genes. Values are expressed as mean ± SEM. *n* = 6 per group. **^#^*P* < 0.05, ^##^*P* < 0.01 vs. CON group; *P < 0.05, *P* < 0.01 vs. LPS group (two-way repeated-measures ANOVA for behavioral data; one-way ANOVA for others).

To elucidate the potential mechanism underlying the anti-inflammatory effects of REA, we investigated its impact on the MAPK and NF-κB signaling pathways, which are critically involved in regulating inflammatory responses. Western blot analysis revealed that LPS stimulation robustly increased the phosphorylation levels of key signaling proteins, including p65 (a subunit of NF-κB) and p38 (a MAPK kinase), in BV2 microglia compared to the control group. However, intervention with REA markedly suppressed the LPS-induced phosphorylation of both p65 and p38 (Figures 5A–C, *P* < 0.05), indicating that REA effectively inhibits the activation of these two pivotal pro-inflammatory pathways. We further examined the downstream transcriptional output of these pathways by quantifying the mRNA expression of pro-inflammatory cytokines. Consistent with the suppression of upstream signaling, qRT-PCR analysis showed that LPS stimulation significantly upregulated the mRNA levels of TNF-α, IL-6, and IL-1β. REA treatment effectively counteracted this effect, significantly reducing the expression of these inflammatory mediators (Figure 5D, *P* < 0.05). This inhibitory pattern at the transcriptional level aligns with the observed reduction in secreted cytokine proteins, thereby consolidating the conclusion that REA attenuates neuroinflammation by targeting the MAPK/NF-κB signaling axis.

## 4 Discussion

Traumatic brain injury represents one of the most complex challenges in clinical neuroscience, characterized by a primary mechanical insult followed by a prolonged and deleterious secondary injury phase (28). Despite decades of research, current therapeutic strategies remain largely supportive, focusing on symptom management rather than targeting fundamental pathological mechanisms (29). This study provides compelling evidence that Rehmannioside A, a natural iridoid glycoside derived from *Rehmannia glutinosa*, confers comprehensive neuroprotection in experimental TBI through multimodal mechanisms centered on the suppression of microglia-mediated neuroinflammation via the MAPK/NF-κB signaling axis.

Our investigation substantially advances the understanding of REA's therapeutic potential by systematically demonstrating its efficacy across multiple functional domains in TBI. While previous studies had documented REA's benefits in spinal cord injury models (30), its effects in TBI remained largely unexplored. We established that REA treatment not only accelerated the recovery of basic neurological functions as measured by mNSS but also significantly preserved higher cognitive functions, evidenced by reduced escape latency and increased platform crossings in the Morris water maze. These behavioral improvements were underpinned by robust histopathological evidence showing attenuated cerebral edema and significant preservation of neuronal architecture in vulnerable hippocampal regions. The convergence of functional recovery and structural preservation observed in our study represents a more comprehensive demonstration of neuroprotection than previously reported for REA, suggesting its potential to address the multifaceted nature of TBI pathology. Furthermore, our dose-response established 40 mg/kg as an effective therapeutic dose, providing crucial preclinical data for future translational studies.

The transition from phenomenological observation to mechanistic insight represents a significant advancement in understanding REA's actions. We hypothesized that the neuroprotective effects observed *in vivo* were mediated through specific modulation of microglial activation, a central driver of post-TBI neuroinflammation (31). Our data provide compelling support for this hypothesis across multiple experimental levels. In the murine TBI model, REA treatment significantly reduced the activation density of IBA1-positive microglia in peri-lesional areas. More importantly, we employed a reductionist approach using LPS-stimulated BV2 microglia to directly demonstrate REA's cell-autonomous effects on microglial inflammatory responses. Our integrated approach, combining *in vivo* and *in vitro* models, provides unequivocal evidence that microglia represent a primary cellular target for REA's anti-inflammatory actions. The consistent suppression of TNF-α, IL-1β, and IL-6 at both protein and transcriptional levels across both experimental systems indicates that REA acts through fundamental regulation of the inflammatory cascade rather than merely symptomatic suppression.

The most significant mechanistic insight from our work lies in the elucidation of REA's simultaneous targeting of both MAPK and NF-κB signaling pathways. These pathways represent convergent nodes in the intracellular signaling network that translates diverse inflammatory stimuli into coordinated transcriptional responses (32). Our findings demonstrate that REA effectively suppresses the phosphorylation of p65 NF-κB and p38 MAPK in activated microglia, thereby interrupting the downstream cascade that leads to pro-inflammatory gene expression. The simultaneous inhibition of these two pivotal pathways represents a distinct advantage over single-target approaches, as it potentially disrupts multiple amplification loops in the neuroinflammatory network (33). This dual inhibition mechanism may explain the potent suppression of multiple cytokines observed in our study and positions REA as a multi-target therapeutic agent capable of addressing the complexity of neuroinflammatory signaling in TBI.

When contextualized within the current landscape of TBI therapeutics, REA's approach offers several distinctive advantages. Unlike conventional anti-inflammatory agents such as corticosteroids, which have demonstrated limited efficacy and significant side effects in TBI (34), REA appears to modulate rather than broadly suppress neuroimmune responses. Our data suggest that REA specifically targets maladaptive inflammatory signaling without completely abolishing microglial functions, potentially preserving their beneficial roles in debris clearance and tissue repair (35). This nuanced immunomodulation contrasts with the blunt immunosuppression associated with many existing anti-inflammatory strategies and may explain the favorable safety profile observed in our toxicological assessments. Furthermore, as a natural compound with known traditional use, REA may offer better translational potential compared to novel synthetic agents with uncharacterized long-term safety profiles.

Beyond its specific implications for TBI treatment, our study offers insights relevant to a broader spectrum of neuroinflammatory conditions. The demonstrated efficacy of REA in modulating microglial activation states through MAPK/NF-κB pathway inhibition suggests potential applications in other CNS disorders where neuroinflammation features prominently, including Alzheimer's disease (36), multiple sclerosis (37), and Parkinson's disease (38). The multi-target nature of REA aligns with contemporary understanding that complex diseases often require multi-faceted therapeutic approaches rather than single-target interventions (39). Our detailed elucidation of REA's mechanism provides a template for how natural products with pleiotropic effects can be systematically evaluated for therapeutic potential in complex neurological disorders.

While our findings provide substantial insights into REA's neuroprotective mechanisms, several limitations warrant consideration. The complex cellular crosstalk in the TBI environment involves not only microglia but also astrocytes, oligodendrocytes, and vascular components, which were not fully explored in the current study (40–43). Future investigations should examine REA's effects on these additional cell types to obtain a more comprehensive understanding of its cellular targets. Although we identified the involvement of NF-κB/MAPK pathways, the initial molecular target of REA—potentially upstream of TLR4 or other pattern recognition receptors—remains to be elucidated. Additionally, while we documented reduced pro-inflammatory markers, a more systematic analysis of microglial polarization using a broader panel of M1 and M2 markers would provide deeper insights into REA's immunomodulatory capacity (44).

Future research should prioritize several directions: first, the identification of REA's primary molecular target through techniques such as affinity chromatography or drug affinity responsive target stability (DARTS) assays; second, the evaluation of REA's therapeutic window in TBI models to guide potential clinical translation; third, the investigation of REA's effects on long-term recovery and cognitive outcomes in chronic TBI models; and finally, the exploration of potential synergistic effects when REA is combined with other neuroprotective strategies.

## 5 Conclusion

In summary, our study provides compelling evidence that REA is a promising therapeutic candidate for TBI. We have systematically characterized its efficacy in improving functional and histological outcomes and, most innovatively, delineated a novel mechanism of action involving the dual suppression of the MAPK/NF-κB pathways in microglia. These findings not only deepen the understanding of REA's neuroprotective pharmacology but also highlight the value of multi-target natural compounds in treating complex conditions like TBI.

## Data Availability

The datasets presented in this study can be found in online repositories. The names of the repository/repositories and accession number(s) can be found in the article/Supplementary material.
